# Broadband Dielectric Spectroscopic Characterization of Thermal Stability of Low-k Dielectric Thin Films for Micro- and Nanoelectronic Applications

**DOI:** 10.1149/2.0141709jss

**Published:** 2017-08-29

**Authors:** Christopher E. Sunday, Karl R. Montgomery, Papa K. Amoah, Yaw S. Obeng

**Affiliations:** Engineering Physics Division, Physical Measurement Laboratory, National Institute of Standards and Technology, Gaithersburg, Maryland 20899, USA

## Abstract

In this paper, we discuss the use of broadband microwaves (MW) to characterize the thermal stability of organic and hybrid silicon-organic thin films meant for insulation applications in micro- and nanoelectronic devices. We take advantage of MW propagation characteristics to extract and examine the relationships between electrical properties and the chemistry of prototypical low-k materials. The impact of thermal anneal at modest temperatures is examined to shed light on the thermal-induced performance and reliability changes within the dielectric films. These changes are then correlated with the chemical changes in the films, and could provide basis for rational selection of organic dielectrics for integrated devices.

Semiconductor manufacturers have been shrinking transistor size in integrated circuits (IC) to improve chip performance and energy efficiency. Consequently, interconnect delay has become a bottle neck for advanced very-large-scale integration (VLSI) systems, and new materials are needed for technology nodes beyond 22 nm to address this problem.^1^ The introduction of emerging materials comes with a myriad of reliability issues arising primarily from the thermomechanical properties of the new materials. In contrast with conventional silicon oxides, low- and ultra-low dielectric constant (i.e., low-k and ULK) materials, with tunable dielectric constants, tend to have decreased thermal performance and diminished mechanical properties.^2–4^ Due to the differences in the thermal, chemical and mechanical properties of these materials, stress can build up within the integrated systems that use these new materials, and may cause mechanical damage. The low-k dielectric materials have different coefficients of thermal expansion from the metals they clad, which leads to the formation of strong local tensile stresses and thermal issues, particularly if the device is thermally cycled in the broad processing temperature range of 25°C to 450°C. With many of the emerging dielectric materials, repeated thermal cycling results in molecular and structural changes, such that the lowest-lying dielectric layer may not have the same properties as the back-end-of line dielectrics of the same composition which have been exposed to fewer thermal cycles. These material changes can have significant impact on the performance and reliability of the integrated system because the propagation velocity of electromagnetic waves in such systems depends on the dielectric properties of the materials of construction.^5^

The design of low- and ultra-low dielectric constant materials involves balancing competing molecular properties.^6,7^ The chemistry of these materials plays a crucial role in determining the performance and reliability of the emerging electronic devices, especially in low voltage devices.^8^ In general, materials composed of molecules with low polarizabilities make better low-k materials. However, these low polarizability molecules suffer from poor adhesion to surfaces because they are quite inert and do not form interfacial films to the target substrates, and, in addition, are mechanically too soft.^9^ Based on materials research and engineering, porous hydrogen-rich amorphous carbon-doped organo-silicon-oxide low-k films (SiOC:H), with uniformly distributed micropores,^4^ have emerged as the material of choice for most commercial back-end-of-line interconnect applications.^10^ Better insights into the fundamental properties of the emerging dielectric are needed to address the performance and reliability gaps of advanced electronic devices.^11^ Among the many vexing issues, there is a need to understand the physics of failure of these new classes of dielectrics including how they breakdown under thermal and electrical stress.^12^

Historically, the matching of low-k materials to applications has been guided by engineering information and intuition.^13^ Since the electrical performance of the materials is the key issue in the evaluation of candidate low-k materials,^4^ it is a common industry practice to screen different dielectric materials by their area-normalized capacitance (i.e., capacitance density), using techniques such as impedance measurements on metal-insulator-metal (MIM) capacitors, interline capacitance, dielectric strength, and breakdown voltage. These extrinsic measurement quantities, which relate to the dielectric constant (k) and the thickness of the dielectric material, are evaluated assuming that the test devices are ideal,^14^ and that any statistical variation is a consequence of the limited technological process control.^4^ However, as we have shown elsewhere, the materials are imperfect, particularly with respect to their chemical and physical properties.^13,14^ For example, water absorption during processing can drastically change the electrical properties of dielectrics. Numerical simulations show that for some low-k materials, about 0.4 volume percent chemisorbed moisture can induce up to a 17–23 percent increase of the dielectric constant.^15^ While the current materials engineering and evaluation approaches are reasonable, and have resulted in commercially successful products, the materials selection may result in sub-optimal choices that cannot be used in future technology nodes. Thus, further understanding is required to extend the current knowledge into emerging nanoelectronics.

The thermal stability of many low-k materials depends on the details of the deposition process, the processing conditions (e.g., precursors, RF power, ambient pressure, temperature and humidity), and the fundamental structure of the polymer backbone. The structure of the polymer backbone determines the low-k materials’ decomposition temperature, degradation rate and mechanism. It has been demonstrated that optical photons (~4.9 eV (254 nm)) synergistically act with molecular oxygen and water to cleave Si-CH_3_ bonds in porous polyhydral oligomeric silsesquioxane (POSS) low-k (p-SiCOH) films.^16^ In the case of plasma enhanced chemical vapor deposited (PECVD) fluorocarbon films, the thermal decomposition occurs over a broad range of temperatures. However, there is a critical temperature (depending on the stereochemistry of the polymer backbone), beyond which, the slowest step in the thermal decomposition reaction is dependent on the diffusion of decomposition by-product materials out of the film. Below the critical temperature, the decomposition of the films is reminiscent of the decomposition of bulk teflon, and the activation energy is commensurate with typical C-C bond strength.^9^ The processing environments can also significantly change the physical and electrical properties of the dielectric materials from the native properties observed in test structures.^17^ Residual free radicals in the polymer backbone during deposition react with oxygen in the aerated ambient to form unstable intermediates (e.g., peroxyl groups) thus forming thermally unstable weak linkages in the polymer backbone. Such thermal degradation may cause the dielectric to break down prematurely.^19,28^

We are interested in metrology for the accurate quantification and mechanistic understanding of the reliability issues associated with the back-end-of-the-line (BEOL) metallization in emerging nanoelectronic devices.^18–25^ The objective of this work is to shed some light on the thermal stability of prototypical emerging low-k dielectric films, as a function of material type and deposition methods, and to relate the thermally-induced changes in chemical properties that occur within the films. In this work, timed thermal storage at modest processing temperatures (200°C, under nitrogen) was used as the perturbation stress to examine the impact of heat on the electrical behavior of un-patterned thin films of the low-k material samples on silicon substrates. We take advantage of changes in the microwave insertion loss due to attenuation^26^ to extract and examine the relationships between chemical and electrical properties.^27^ Other workers have also used impedance spectroscopy to study dielectric loss in low-k capacitor devices, and have concluded that the impedance spectroscopy technique has the potential to provide insights into the physics of failure during accelerated time-dependent dielectric breakdown (TDDB) testing for dielectrics.^28^ It is anticipated that the results from on-going efforts will provide bases for rational selection of organic dielectrics for future integrated devices.

## Theoretical Background

Microwaves (MWs) can be transmitted, absorbed or reflected depending on the type of material they interact with. When MWs penetrate and propagate through a dielectric material, the radiation induces polarization within the material that is equal to the dipole moment per unit volume,^29^ and the internal field generated within the effected volume induces translational motions of free or bound charges such as electrons or ions, and rotates charge complexes such as dipoles.^30^ These motions result in MW energy loss in the material. In dielectric (electrically insulating) materials, the absorption of microwaves is related to the material’s complex permittivity, ε = ε_0_(ε^I^ − *i*ε^II^), where ε_0_ is the permittivity in free space (8.86 × 10^−12^ F/m), the real part, ε^I^, is the relative dielectric constant, and the imaginary part, ε^II^, is the effective dielectric loss factor. The microwave absorption is maximized through large dielectric repolarization;^31^ thus, analyses of the electromagnetic properties of materials, such as complex permittivity and permeability should provide insights for understanding the underlying mechanism of microwave signal losses in the low-k materials. The frequency-dependence of dielectric properties will provide important information on the materials’ chemistry.

**Figure 1:**
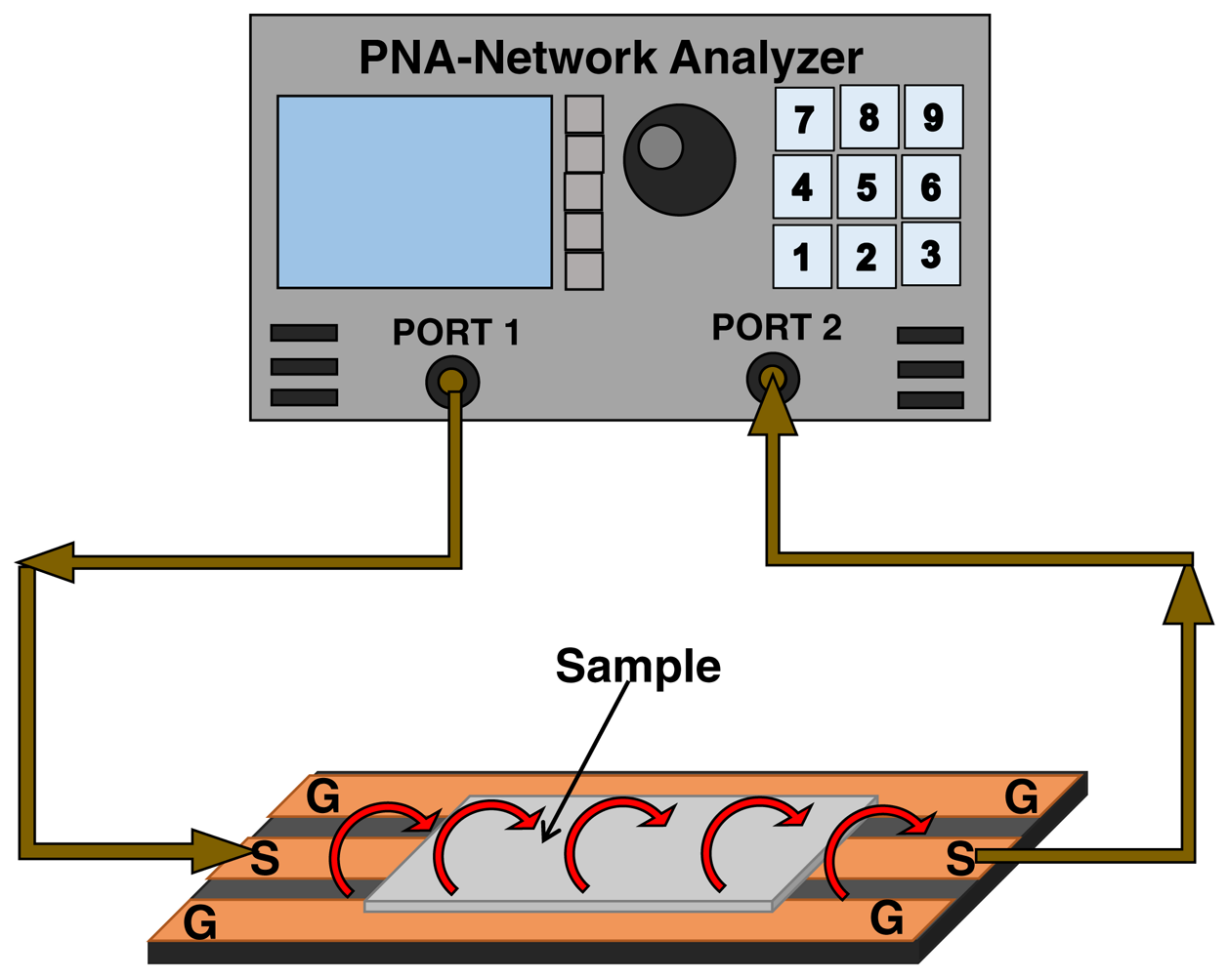
Illustration of a sample placement on the waveguide for measurement.

As shown in Figure 1, the samples were placed on ground-signal-ground (GSG) waveguides comprised of aluminum traces on a PCB board, and were probed with evanescent microwaves. The GSG waveguide can be considered to be a uniform transmission line. With the sample on the waveguide, it can be modeled as a homogenous dielectric filled waveguide,^26^ such that the wave propagation constant (Γ) can be defined as:^32^

[1] Γ=α+jβ

While in principle, both dielectrics and conductors contribute to the energy loss, the loss tangent of the device-under-test (DUT) is sufficiently large such that the energy loss into the dielectric far exceeds conductor loss. Thus, we attribute all the insertion losses to energy absorption into the dielectric film, and use the attenuation constant as a measure of MW absorption into the film. The attenuation constant (α) can be defined as:

[2] $$\alpha =\frac{k^{2}\tan\delta }{2\beta }$$

where $k=\beta =\omega \sqrt{u_{0}\varepsilon_{0}\varepsilon_{r}}$ and *tan***δ** = ε^II^/ε^I^, and tanδ is the loss tangent which signifies the inherent dissipation of electromagnetic energy as the wave propagates in the material.

Mechanistically, the attenuation represents the MW signal losses due to the reorientation of electric dipoles. The attenuation is related to the group refractive index (n_g_), and any changes in attenuation constant give us indications of changes in the chemical and electrical properties of the dielectric material matrix, especially when polar defects are generated during the experiments. In the current study, the attenuation spectra can be correlated to the repolarization of specific functional groups and dangling bonds. Hence, we used the attenuation constants to monitor and quantify the impact of modest heating on different classes of prototypical low-k materials.

## Experimental

### Materials

Two fundamentally different types of low-k materials^6^ were evaluated in this work:

- Homogeneous single component non-porogen Bakelite-type polymer (k = 2.8–3.0, Sample A). It derives its low-k properties primarily from the lack of polar bonds. However, the dielectric constants of such materials may be tuned by the addition of porosity to the matrix.
- Hybrid porous carbon-doped inorganic oxides (p-SiOCH, POSS with Si-C bonds, k< 4, Samples B through F). These are open-cage inorganic poly(methyl silsesquioxane), with structures reminiscent of conventional PECVD SiO_2_, with terminating Si-CH_3_ groups.^15,33^ Where the pendant group is thermally labile, the material can be deliberately thermally degraded to generate porosity which can be used to tune the dielectric constant to as low as 2.83.^34^ Sample B is a fully cured, porous POSS (R = CH_3_, without −OH), deposited by spin-on (SOD) technique from liquid precursors to create films with elastic modulus of 3.4 GPa. On the other hand, samples C and D are both POSS (R = CH_3_) films deposited by chemical vapor deposition (CVD) technique from gaseous organosilane precursors in the presence of oxygen.^35^ Samples F and E are also MSQ POSS materials, but these were further processed with simulated pattern definition plasma etch, simulated ashing and cleaning. While F was deposited by PECVD, E was deposited by SOD spin-on techniques.

The materials studied in this paper are prototypes currently in development, and were obtained as thin films on silicon substrates from a number of sources. They were used “as-received” without any further modification. The technical data provided by the material suppliers are compiled in Table I. The test samples were stored in a N_2_-purged box at laboratory temperatures for several months before use. During the studies, the samples were stored at 200°C in a N_2_-purged pre-heated box oven for up to 9 hours and were removed intermittently for microwave (MW) and Fourier transform infrared spectroscopy (FTIR) measurements. A storage temperature of 200°C was chosen to represent typical processing temperatures in through-silicon via (TSV)-interconnect integration flow. All the samples were approximately 1000 nm thick.

### Microwave measurements

The electrical properties of the thin films were evaluated through evanescent microwave attenuation, by placing the samples on ground-signal-ground (GSG) waveguides comprised of aluminum traces on a PCB board with teflon retaining posts, for measurement as shown in Figure 1. This setup allows the samples (of the same size) to be positioned consistently with minimal effect to the output. The samples were probed with Teledyne LeCroy SPARQ-3004e^+ a^ (Teledyne LeCroy, Chestnut Ridge, NY) signal integrity network analyzer, using calibrated cables.

The reproducibility of the MW measurement technique was evaluated by using well characterized dielectric materials: a quartz plate, a thin film of ozone-TEOS (tetraethyl orthosilicate) (HARP, Applied Materials, Santa Clara, CA),^36^ polymethylmethacrylate (PMMA and polystyrene (PS) deposited on silicon substrates. Room temperature MW and FTIR spectra were measured at predefined intervals during the high temperature storage. The samples were cooled in a desiccator before measurements. The reported attenuation constant data were extracted from the insertion loss (S21) scattering parameters through analysis with MatLab+ software (The MathWorks, Inc., Natick, MA).

## Results and Discussion

### Attenuation in model systems

The low-k materials studied in this work fall into two main categories, organic-inorganic silsesquioxanes, and organic Bakelite-based polymer materials. A quartz plate and SACVD Ozone-tetraethylorthosilicate thin film on silicon substrate (HARP) were used as models for the hybrid organic-inorganic, while thin films of polymethylmethacrylate (PMMA) and polystyrene (PS) thin films on silicon substrate were used as models for the organic low-k materials.^37^

**Figure 2:**
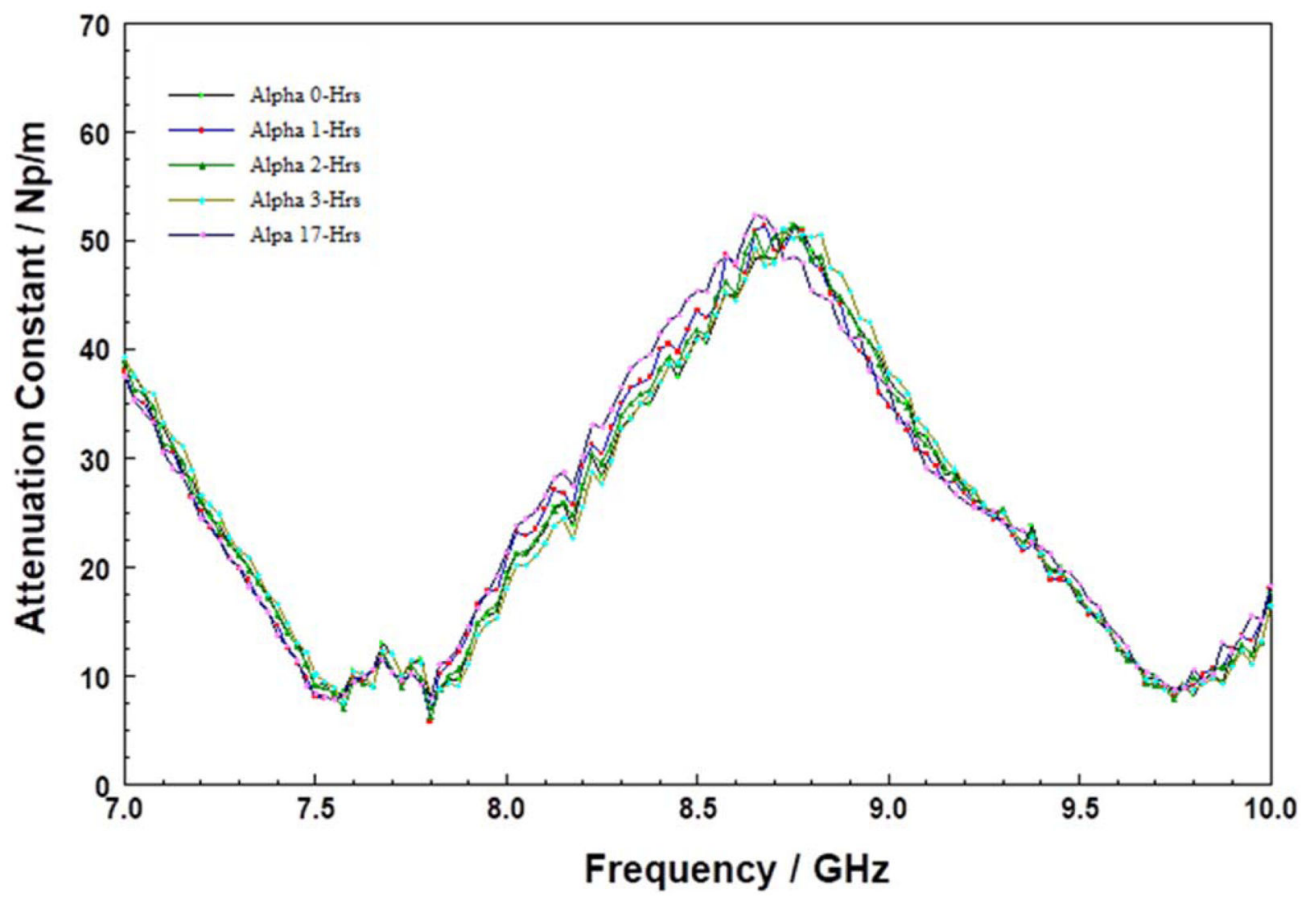
Attenuation constant spectra of the Quartz plate substrate stored at 200°C for five different durations, in the 7 to 10 GHz.

Figure 2 shows the attenuation constant of freshly cleaned quartz, as a function of storage time at 200°C, over a 9-hour period. Inspection shows that the attenuation constant of the quartz benchmark appears to be invariant with heating time. Similarly, the corresponding FTIR spectra (not shown here) did not show any changes over the storage duration. This data set shows the inherent reproducibility of our physical measurements, as well as the sensitivity of the measurements to changes in material chemistry.

**Figure 3:**
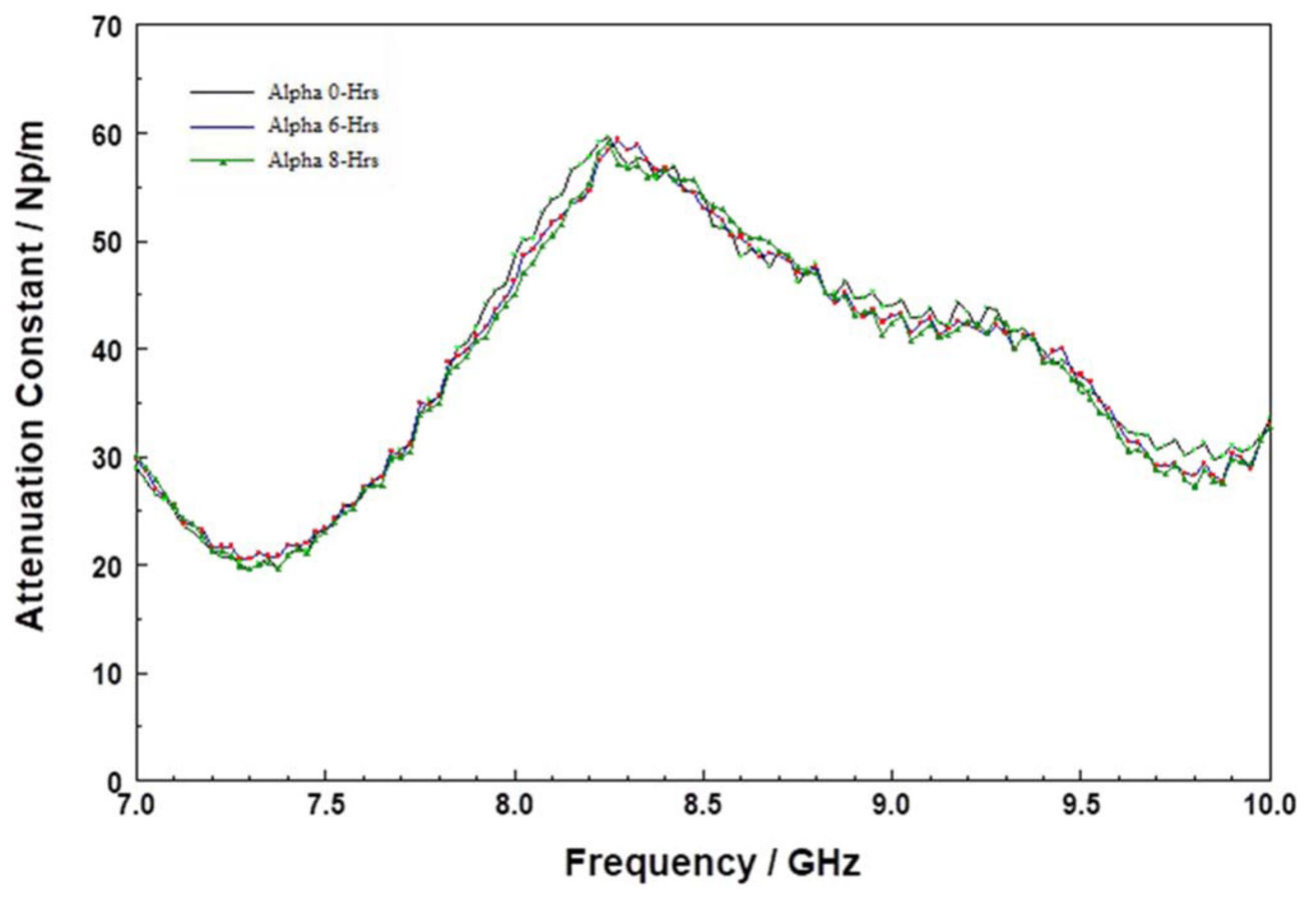
Attenuation constant spectra of HARP thin film on silicon substrate stored at 200°C for 0, 6, and 8 hours in the 7 to 10 GHz range.

Figure 3 shows the attenuation constant spectra of “time-aged” HARP as a function of storage time at 200°C, over a 9-hour period. As with the quartz data, Figure 2, the attenuation constant for the HARP film did not change appreciably in these experiments. The film we used for benchmarking was chemically stable and was stored in a N_2_ purged box at room temperature for over three years before use. The invariance of the attenuation constant with the 200°C storage time confirms that either the film is fully cured.^36^

**Figure 4:**
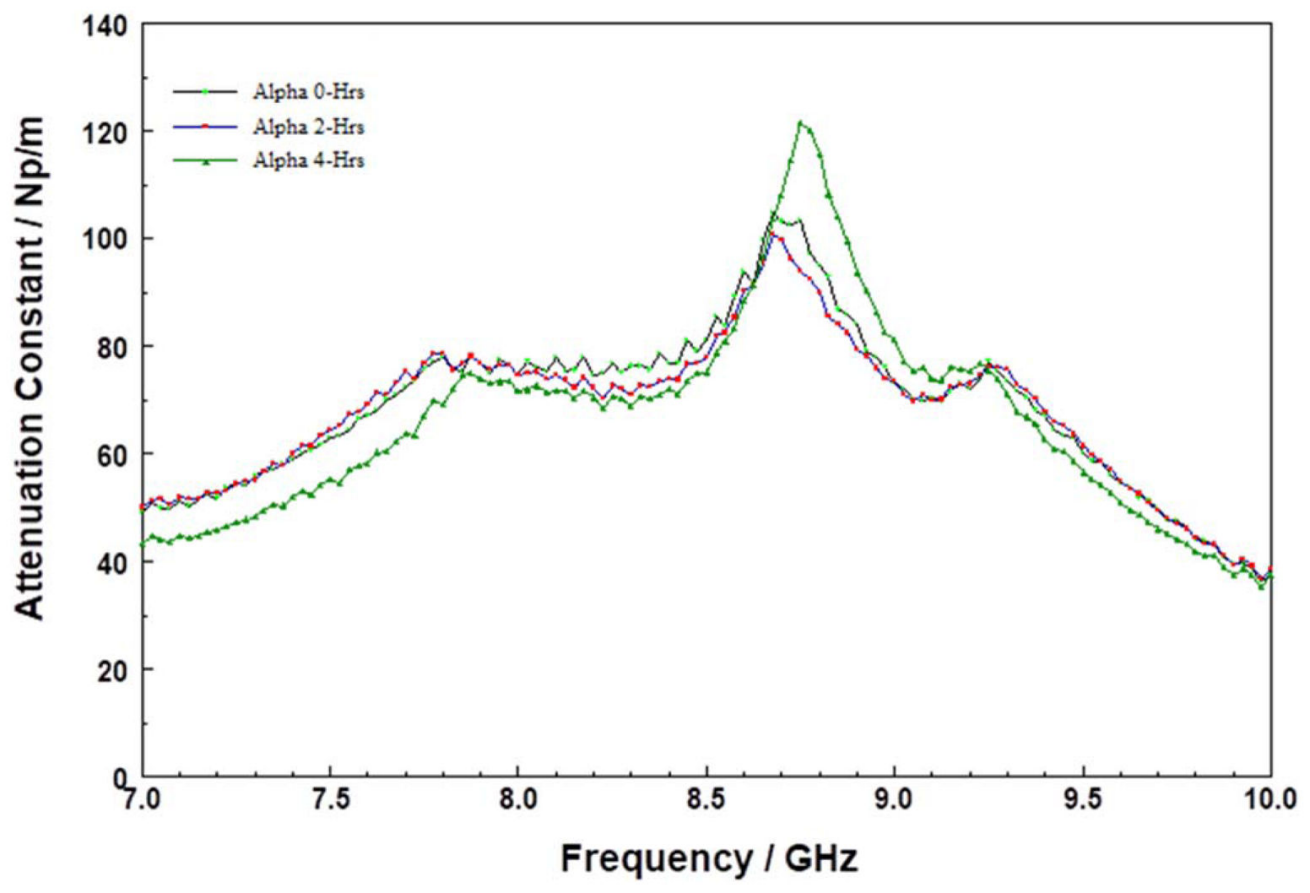
Attenuation constant spectra of PMMA thin film on silicon substrate stored at 200°C for 0, 2, and 4 hours in the 7 to 10 GHz range.

**Figure 5:**
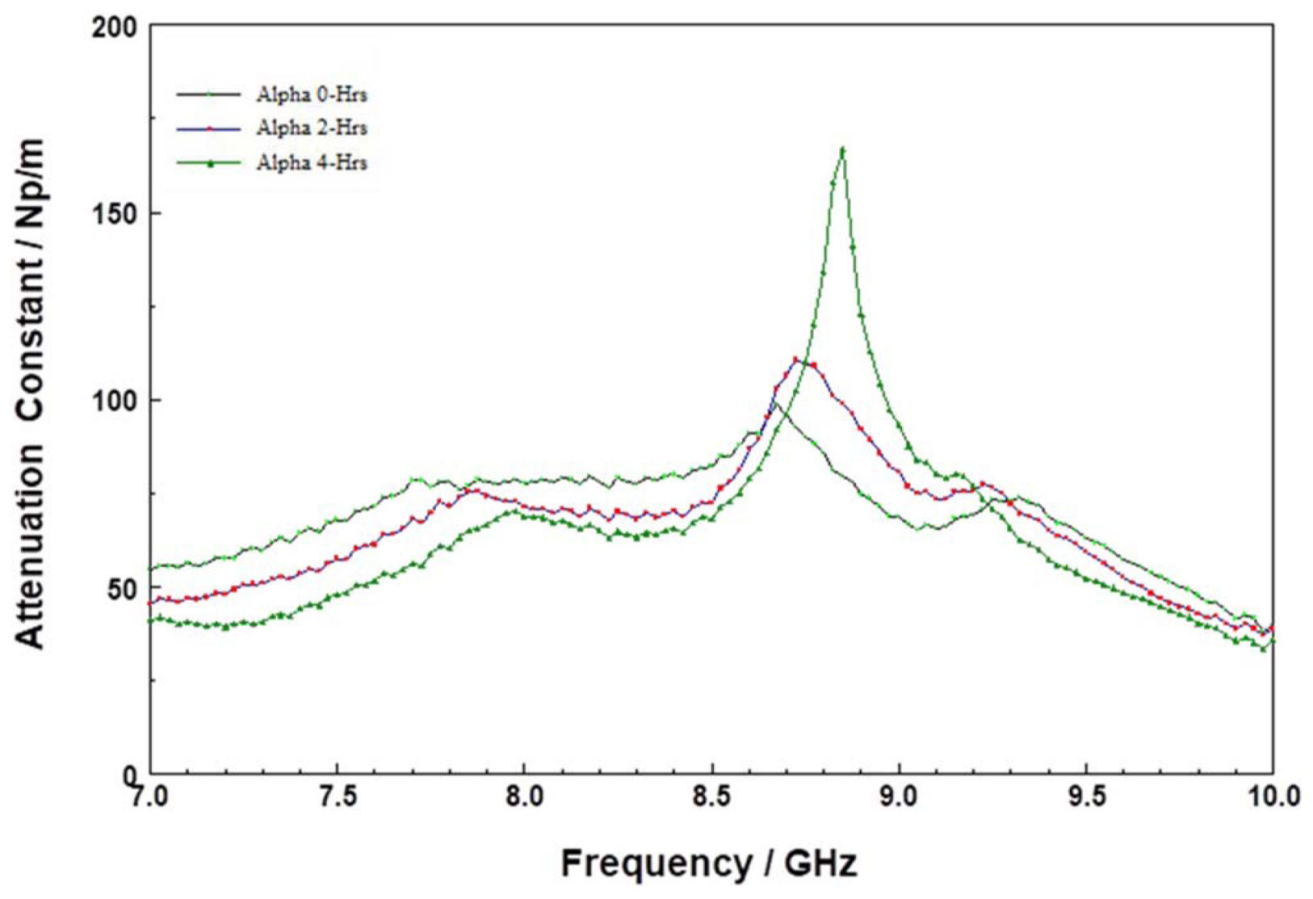
Attenuation constant spectra of PS thin film on silicon substrate stored at 200°C for 0, 2, and 4 hours in the 7 to 10 GHz range.

Unlike the thermally stable inorganic models, both PMMA and PS are known to be thermally unstable. Both PMMA and PS films gradually thermally decompose by random-bond scission, to produce polar dangling bonds.^38^ As shown in Figures 4 and 5, respectively, the attenuation spectra of PMMA and PS thin films changed with storage time as expected. The observed changes are probably due to changes in the nature and distribution of dangling and polar bonds generated from polymer chain scissoring during the storage at 200°C. Both figures show three distinct attenuation constant peaks in the 7 to 10 GHz range, which are indicative of polar aromatic functional groups (e.g., alcohols, aromatic ketones, and amides, etc.^39^). There is a sharp rise in the peak around 8.6 GHz, which suggests an increase in concentration of the MW absorbing species. These observations indicate a depolymerization process, which creates smaller fragments of polymer chains, such that the dangling bonds or moieties are responsible for the MW absorption. These dangling bonds or moieties are free to rotate at higher rates as compared with the starting materials.

PMMA films decompose slowly in the 100–215°C range due to degradation initiated by scission of weak peroxide and hydroperoxide linkages to generate volatile products comprised of mostly methyl-methacrylate (MMA) monomer and ethylformate (HCOOCH_3_).^40^ Head-to-head linkage fracture in the polymer also leads to the production of free radicals, which can participate in further depolymerization at higher temperatures through chain transfer processes.^41^ Similarly, polystyrene films also decompose by prolonged exposure at 125°C^42^ through random chain scission reactions of labile “weak links” within the polymer chains.^43^

### Thermal stability of the bakelite-polymer based low-k material

**Figure 6:**
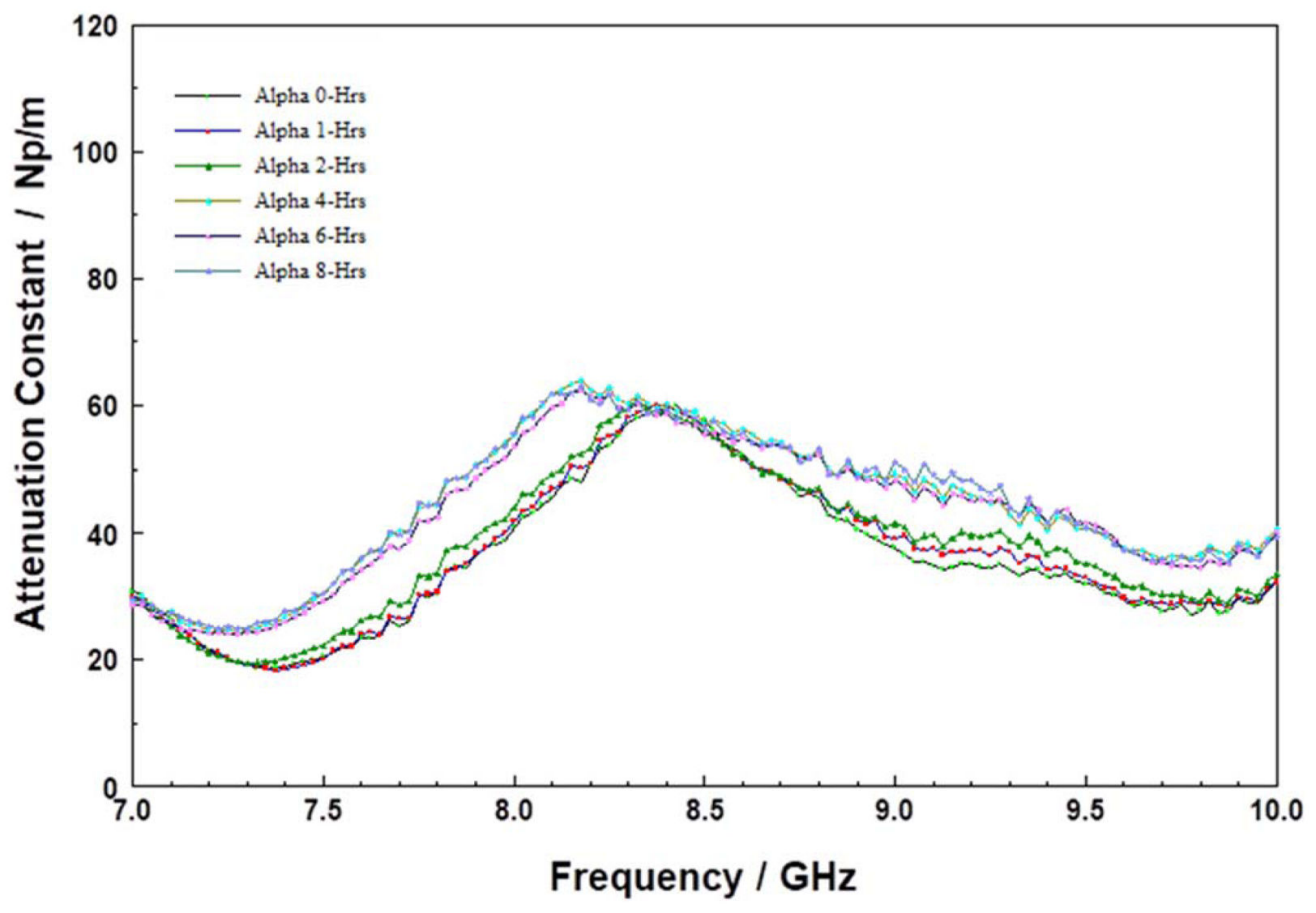
Attenuation constant spectra of A at 200°C for various times in the 7 to 10 GHz range.

Sample A is an bakelite-type (i.e., condensation products of aromatic phenols and formaldehyde) polymer-based low-k material, built on highly crosslinked short chained thermoset phenol/formaldehyde resins polymer architecture.^44^ In Figure 6, the MW attenuation constant spectrum after 4 hours is different from those of the “as-received” and those stored up to 2 hours. The absorption at 8.5 GHz initially did not change for the first 2 hours of storage, but after 4-hours, the absorption shifted to lower frequencies. The isosbestic point at approximately 7.3 GHz suggests that the changes responsible for the MW absorption are related to systematic molecular transformation rather than uncontrolled decomposition. It is noteworthy that the shift in the MW absorption spectra occurred after 4 hours of storage at 200°C, and did not change again with increased storage time. This suggests that material was transformed from a relatively stable form into another form, and that the transformation is either not kinetically driven or has a high activation energy. Heating of bakelite-type polymers to temperatures of approximately 200°C leads to weight loss, which is probably due to depolymerization via methylene bridge loss,^45^ and results in the creation of dangling bonds.^46^ Thus, the shift in the spectrum suggests that while the film did not spontaneously decompose, a rather slow, systematic depolymerization resulted in polar groups being able to move more freely. The exact nature of the thermally-induced transformations in bakelite-type films will be resolved in future work.

### Thermal stability of the POSS based low-k material

The hybrid carbon-doped silicon oxide (POSS SiCOH) materials, belong to a class of organosilicon compounds with the general formula (RSiO_1.5_)_n_, where R is hydrogen or an organic group, such as an alkyl, aryl or any of their derivatives. Depending on the organic character of the precursor and the method of deposition, these films may further react (i.e., condensation in the siloxane network) or decompose, resulting in weight loss, with modest heating. The weight loss involves sublimation of the POSS, together with traces of water and carbon dioxide in competition with macromer homo-polymerization reactions involving -Si-O-Si- condensation, Si–C cleavage, and carbon–carbon chain cracking to produce unsaturated moieties.^47^ At 200°C storage temperature, most of the POSS materials undergo glass transition. In air, oxidation phenomena involving the peroxidation of the alkyl chains and the subsequent fragmentation through classical radical pathways also occur.

**Figure 7:**
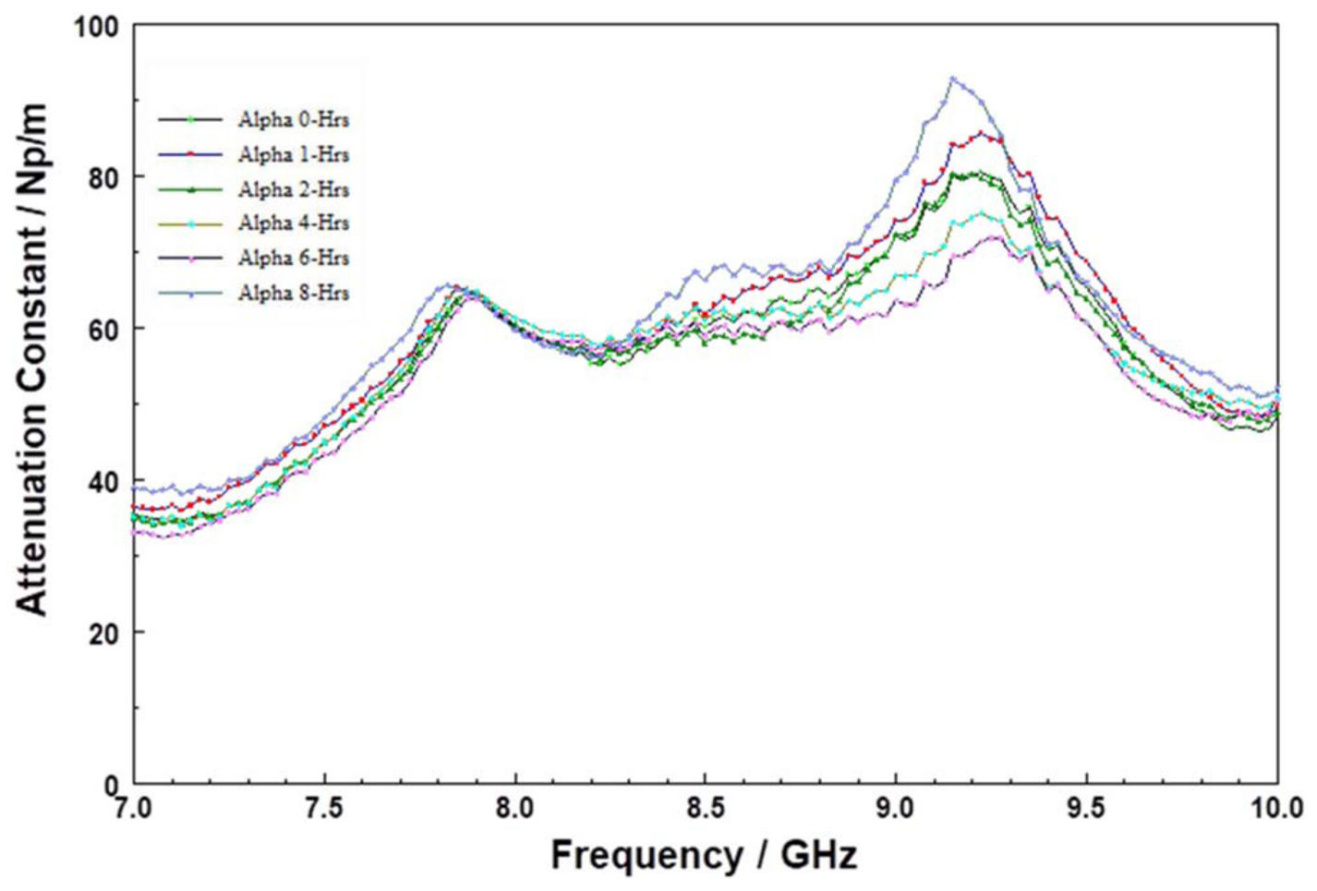
Attenuation constant spectra of B at 200°C for various times.

As shown in Figure 7, the attenuation spectra of the SOD POSS film (sample B) showed peaks at approximately 7.8 and 9.2 GHz, respectively. The resonance peak locations did not change significantly with storage for the first 6 hours. However, for some unknown reason, the peak intensities varied unsystematically, but repeatably over time. A new peak emerges at approximately 8.5 GHz at the 8-hours storage mark, indicating that while the film did not change for the first 6 hours; it began to decompose after approximately 8-hours of storage at 200°C. The evolution of the attenuation peaks suggests that the SOD POSS material (sample B) was reasonably thermally stable under the storage conditions used in this work.

**Figure 8:**
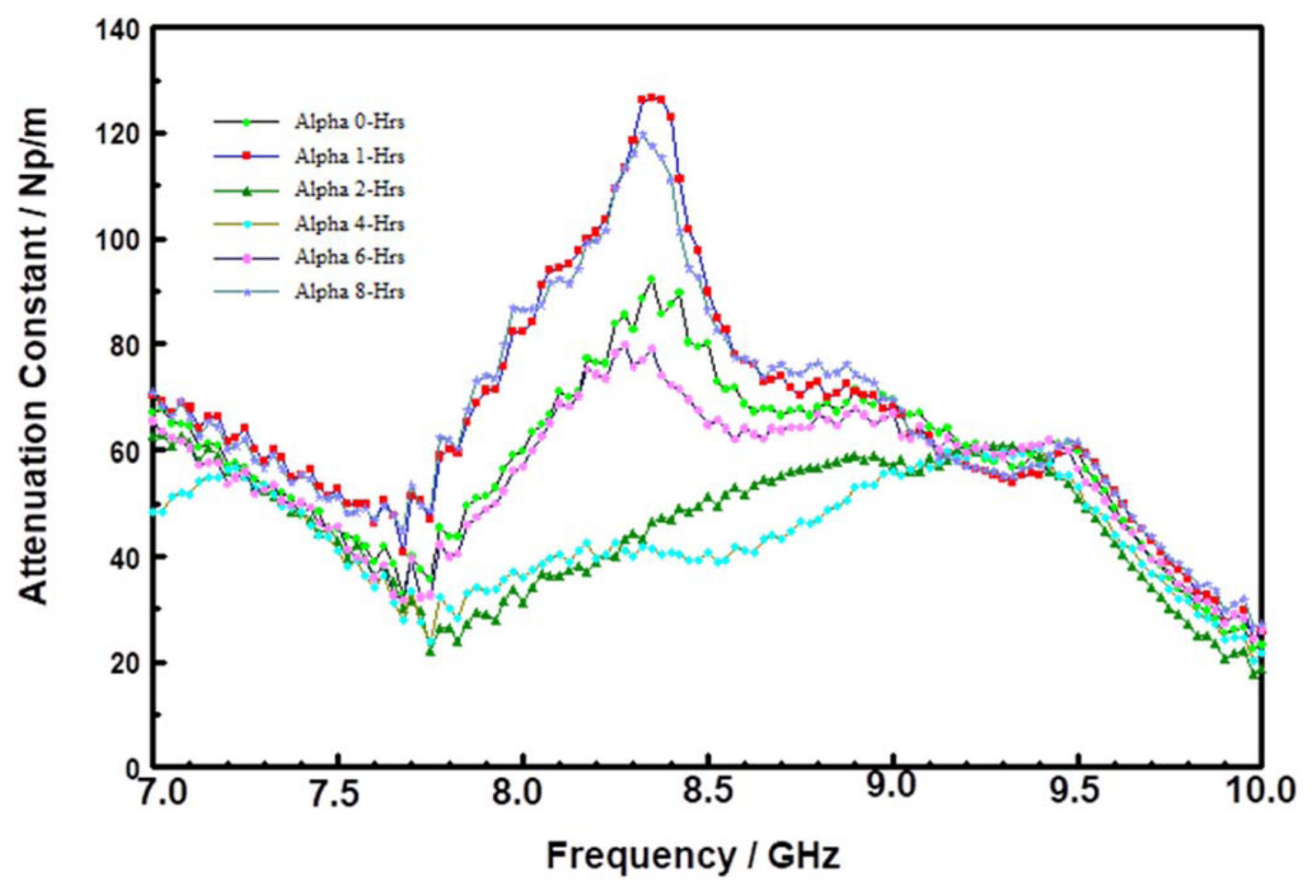
Attenuation constant spectra in the 7 to 10 GHz range of Sample C stored at 200°C for six different durations. The data suggest changing MW absorption with storage time at 200°C.

**Figure 9:**
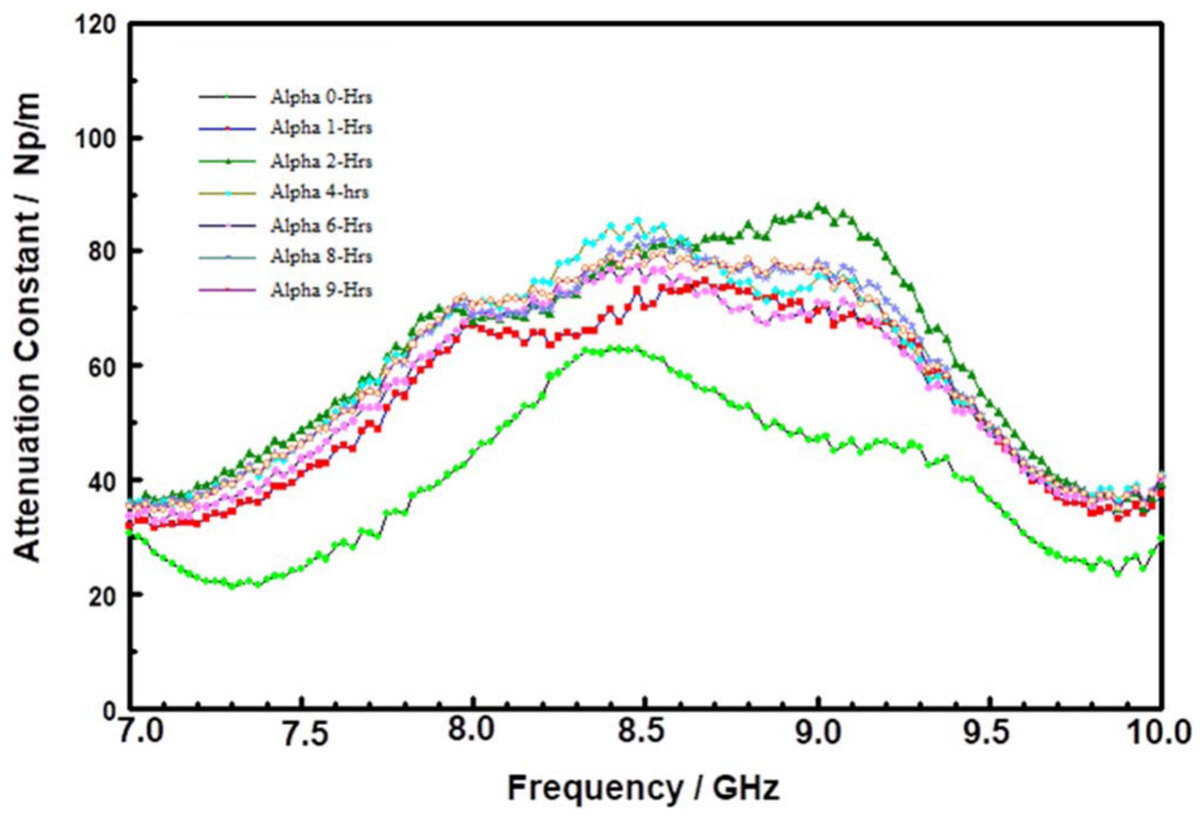
Attenuation constant spectra in the 7 to 10 GHz range of Sample D stored at 200°C for various times.

In contrast, to the SOD POSS film, the attenuation spectra for the CVD films, Samples C (Figure 8) and D (Figure 9), respectively, changed drastically after just one hour of storage at 200°C. Both samples show a broad peak in the 7.5 to 9.0 GHz range. For Sample C, the peak centered at approximately 8.3 GHz, increased in intensity during the first two hours of storage, and then systematically decayed after 2 hours. In the case of Sample D, the peak intensity increased and shifted to higher frequencies. These changes are suggestive of thermal transformation of the initial polar groups in the “as-received films” into some other species. The insertion loss spectra of Sample D (S21 spectra not shown here) showed large losses as a function of storage time; with a huge irreversible drop in MW transmission after 1-hour of storage at 200°C under N_2_. It is tempting to attribute these changes in the CVD SiCOH materials to material degradation because of water desorption.^48^

### Identification of thermal degradation products

In the following discussion, we use FTIR to elucidate the thermal degradation products caused by the 200°C storage. We compare pre- and post-storage FTIR spectra, and mathematically subtract the former from the latter to obtain the difference spectra. Our emphasis is on the difference, and not the individual spectra, which may be noisy. This is an important clarification since we obtained the samples from a variety of sources, and are not privy to the histories of the samples.

**Figure 10:**
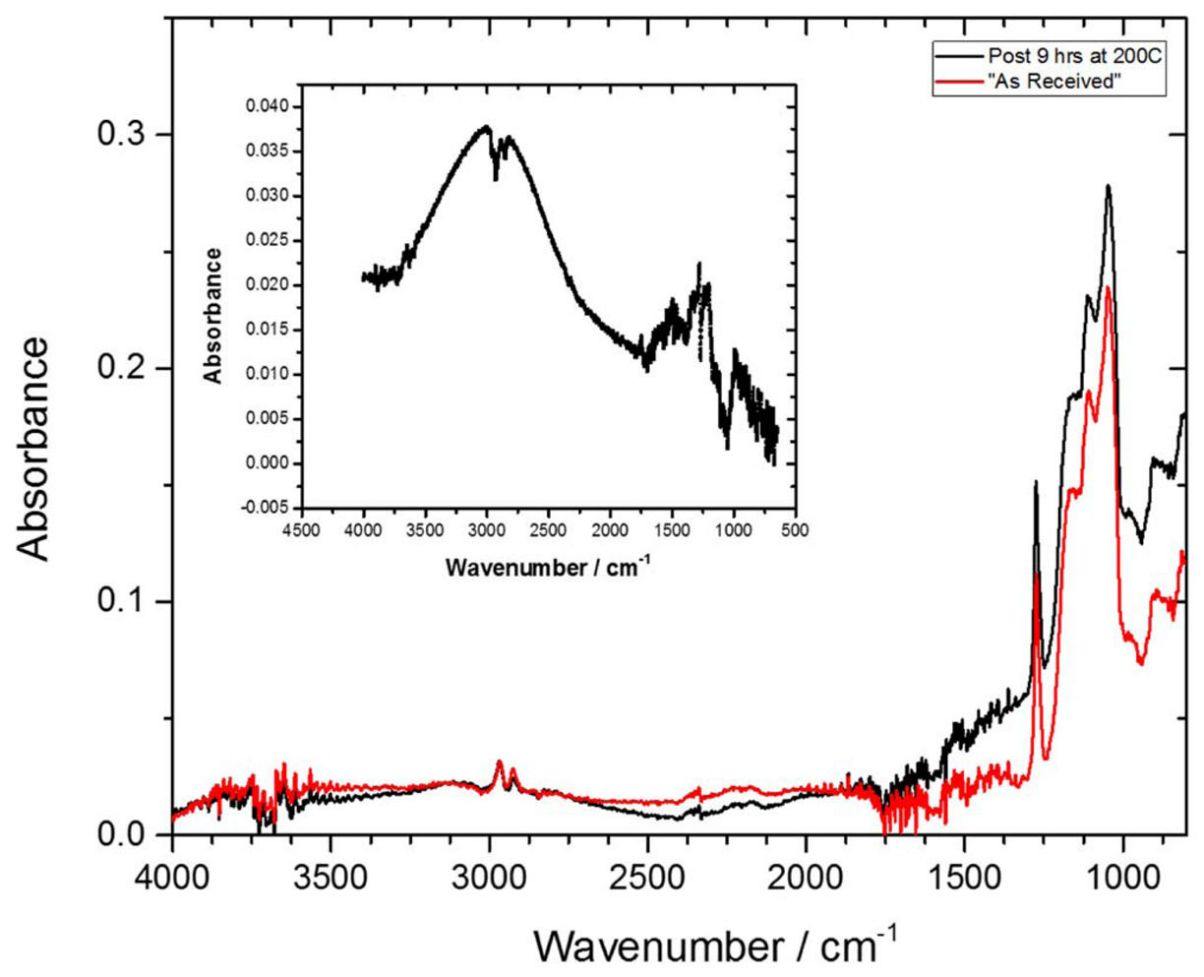
FTIR of Sample D before (red) and after 9-hour (black) storage at 200°C under nitrogen. The insertion is the difference spectrum of the sample before and after 9 hours of storage.

Figure 10 shows the FTIR spectra of “as-received” Sample D and after 9-hour storage at 200°C under N_2_. Both pre- and post-storage FTIR spectra were noisy, but are characterized by a weak broad peak in the 4000–3100 cm^−1^ region, which is indicative of silanols (Si-OH), terminal hydroxide (−OH), or water in the film. This is unusual for a low-k film with an advertised dielectric constant of 2.5, and suggests the “as-received” sample might have been exposed to an oxidizing ambient prior to delivery into our hands. Interestingly, the FTIR data suggests that the film did not absorb additional moisture upon exposure to ambient conditions after 9 hours of storage at 200°C. The difference spectrum, shown as an insert in Figure 10, shows a broad absorption band centered at approximately 3000 cm^−1^ which corresponds to −CH_n_ (n = 1 – 3) vibrations, with additional peaks at 1323, 1277 cm^−1^ and 1206 cm^−1^ which correspond to Si-C stretches.^10^ The broad peak around 3000 is an envelope comprised of 2956 cm^−1^ peak due to s p^2^ -CH_2_ (olefinic), peak at 2920 cm^−1^ due to the sp^3^ -CH_2_ asymmetrical stretching mode, and peak at 2870 cm^−1^ due to sp^3^-CH_3_ symmetrical stretching modes. These peaks are reminiscent of amorphous hydrogenated carbon films.^49,50^ The new peaks observed in the difference FTIR spectrum indicate that the SiOCH backbone underwent a chemical restructure, probably to form segregated hydrogenated carbon domains, when the samples were heated to 200°C. Thus, the observed changes in the MW signal propagation characteristics for sample D after storage at 200°C may be attributed to chemical transformations within the films, which involve the conversion of some intermediate species into amorphous hydrogenated carbon domains and further condensation within the POSS backbone, and not just a physical water loss.

**Figure 11:**
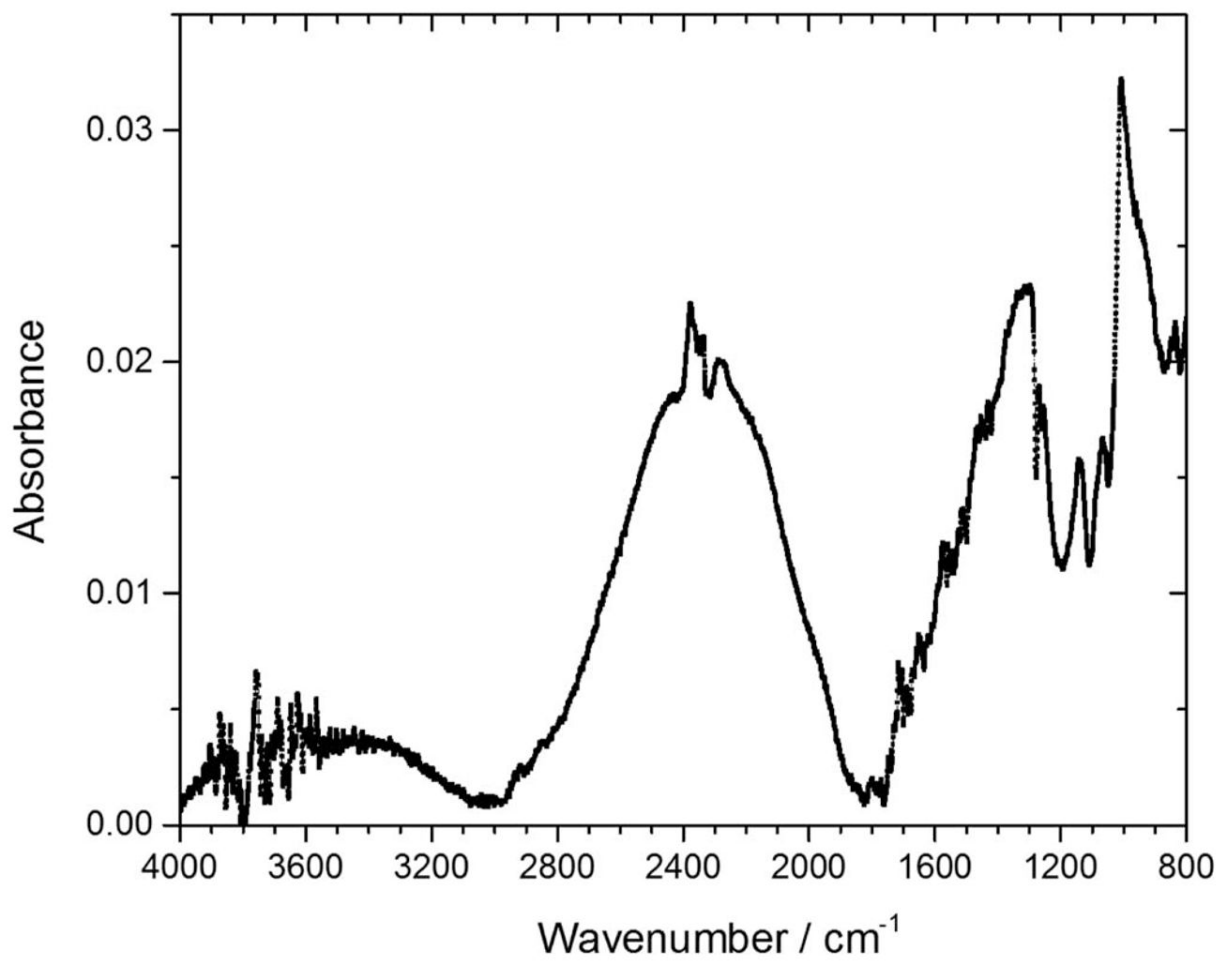
Difference FTIR spectrum of Sample F before and after 4-hour storage at 200°C under Nitrogen.

Samples F and E are both SOD POSS materials that had been further processed with simulated pattern definition plasma etch, simulated ashing, and cleaning. The subsequent processing steps increased the substrate temperature and subjected the dielectric materials to both physical and chemical damage, the degree and nature of which depend on the chemistry and conditions used.^51,52^ Unlike the remarkable thermal stability of the SOD Bakelite-polymer film (B), the attenuation spectra of both Samples F and E (not shown here) are similar to those of CVD POSS (D); they showed significant changes with storage times at 200°C. In the interest of brevity, we will focus only on the difference spectra to illustrate the chemical changes of the film following the high temperature storage. Figure 11 shows the difference FTIR spectrum of Sample F after 4-hours of storage at 200°C under N_2_. Condensation and densification of the polymer network are deduced from the IR vibrations in 1800–1200 cm^−1^ range, due to the incorporation of the Si from Si-CH_3_ groups into the -Si-O-Si- cage structure.^53^ The weak absorption dome-shaped peak in the range of 3100–2600 cm^−1^ is assigned to C–H and O–H, and this peak indicates the creation of new polar functional groups, possibly from the ring opening of carboxylic acid and anhydrides. The three weak peaks at 2855 cm^−1^, 2925 cm^−1^ and 2957 cm^−1^ correspond to sp^3^–CH_2_ symmetric vibrational frequencies, sp^3^–CH_2_ asymmetric vibrational frequencies, and sp^3^–CH_3_ asymmetric vibrational frequencies, respectively.^54^ Again, these new peaks are reminiscent of the hydrogenated carbon domains observed in Figure 10. It appears that in the Si-R bonds in the Sample F fragment, at 200°C, the Si gets incorporated into the siloxane cage structure and the organic fragments aggregate into carbon-rich domains.

**Figure 12:**
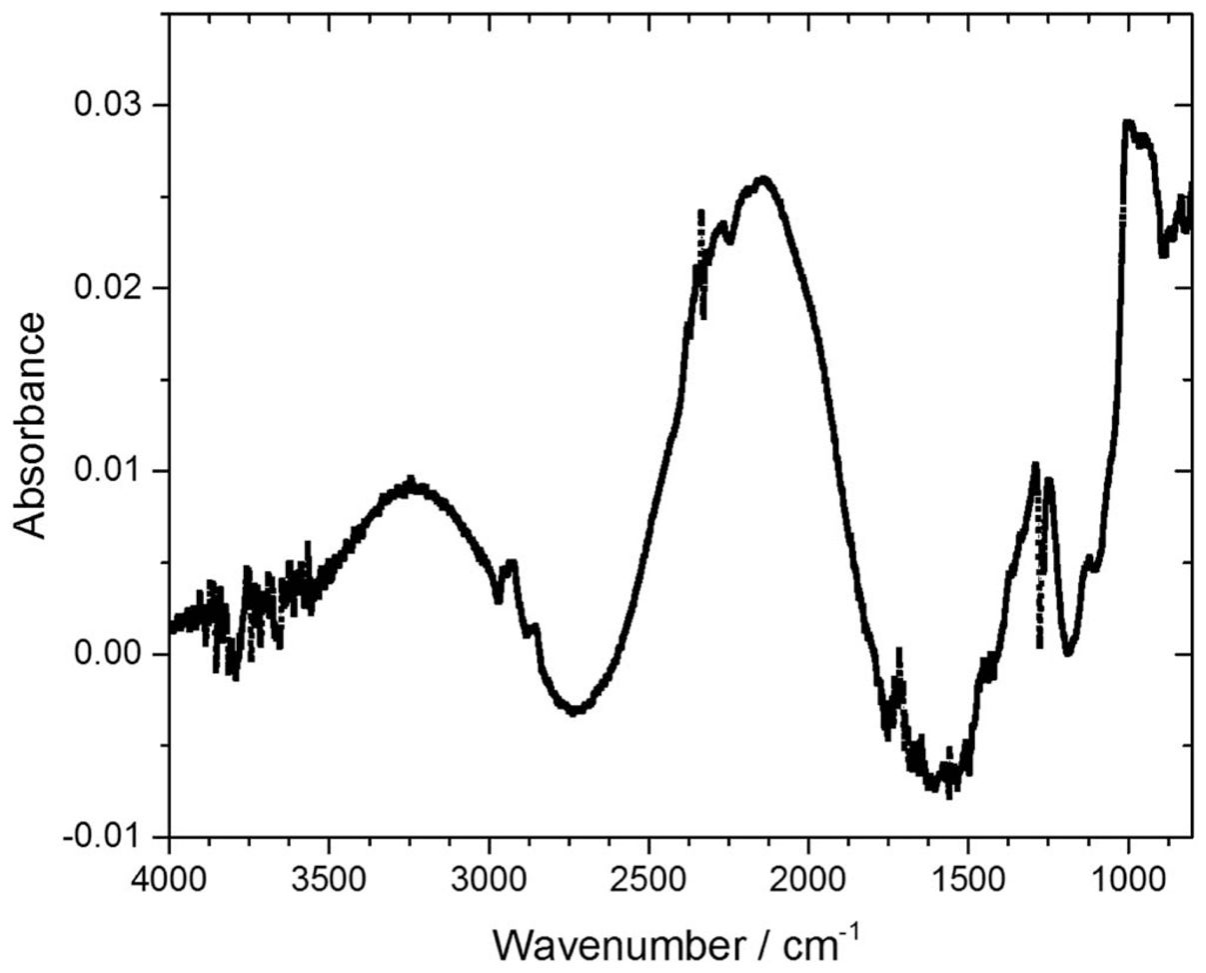
Difference FTIR spectrum of Sample E before and after 4-hour storage at 200°C under Nitrogen.

Figure 12 shows the difference FTIR spectrum of sample E after 4-hours of storage at 200°C under Nitrogen. The changes that occurred in Sample E after the 4-hour storage at 200°C include intensification of asymmetric stretching of -Si-O-Si- due to further condensation within the -Si-O-Si- cage structure network. Strong absorption peaks are also observed at the range 1800–1200 cm^−1^ from organic moieties such as CH_3_, C=O and C=C groups.^55,53^ Again, as with Samples D and F, new peaks were observed in the difference FTIR spectrum, demonstrating that the POSS backbone chemically restructured, probably to form segregated hydrogenated carbon domains when the samples were heated to 200°C. Also, it appears that new polar dangling bonds and condensation reactions occurred, as indicated by the peaks formed in 2200–1000 cm^−1^ range in the difference spectrum for Sample E.

In summary, in all the materials studied, the observed changes in the MW signal attenuation spectra after storage at 200°C may be attributed to chemical transformations within the films, involving, at a minimum, the conversion of some intermediate species into amorphous hydrogenated carbon domains.

### Signal dispersion due to material degradation

The group delay (Γ_g_) which is a measure of how long it takes a signal to travel through a network, hence signal dispersion, is related to the phase constant (β in Equation 1). Operations across a wide band of frequencies, as in high-speed data transfer with ultra-wideband devices, can be constrained by the group delay characteristic of circuit components. If the group delay of devices varies substantially with frequency, the time domain waveform becomes distorted, especially for systems using impulse signals.^56^ The changing nature of the low-k materials could alter the group delay characteristics of devices that incorporate such materials.

**Figure 13:**
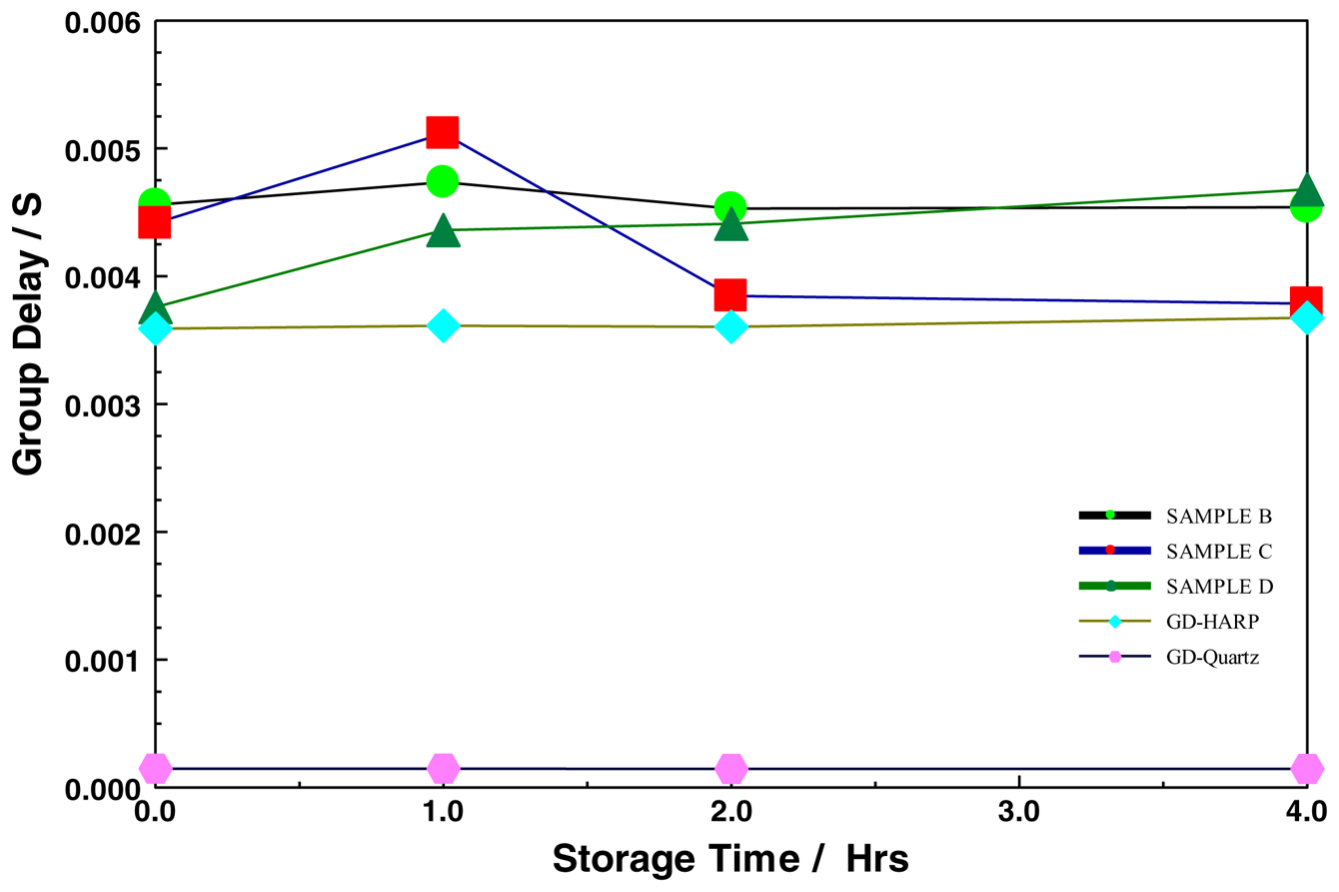
Group Delay of various SiOCOH thin film on silicon substrate, HARP and Quartz stored at 200°C as a function of storage time. The error bars of the group delay are smaller than the symbol size and therefore do not appear in the figure.

**Figure 14:**
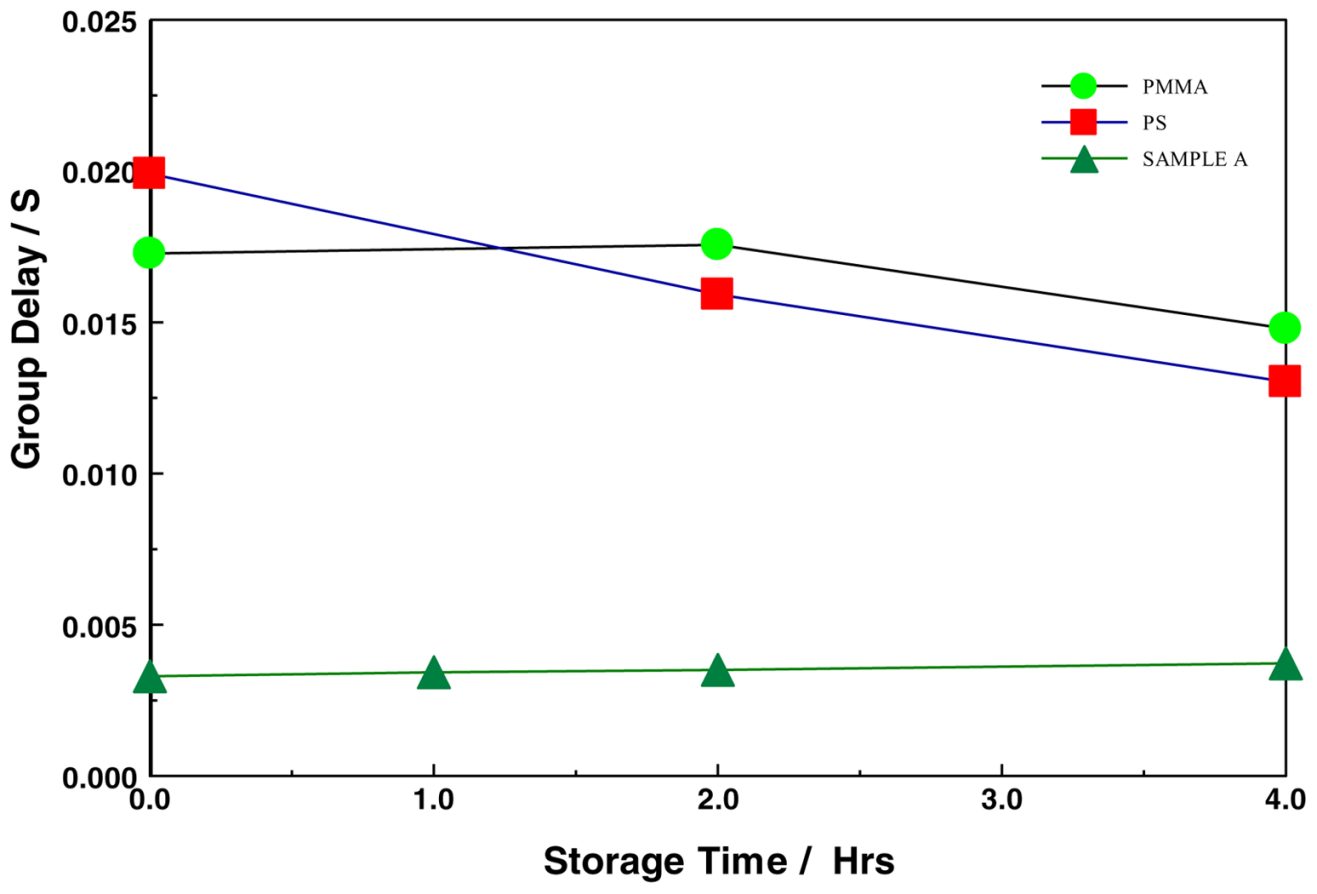
Group Delay of Bakelite-based low-k thin film, PMMA, and PS on silicon substrate, stored at 200°C as a function of storage time. The error bars of the group delay are much smaller than the symbol size.

In this work, the group delay shown in Figures 13 and 14, were extracted from the rate of change of transmission phase shift with respect to angular frequency. Figure 13 compares the S21 group delay of all the POSS SiCOH materials discussed in this paper along with the Quartz and HARP references, while Figure 14 compares the organic Bakelite low-k film to the PMMA and PS films. In Figure 13, the group delays of the quartz and HARP thin films were invariant with storage time at 200°C. On the other hand, the all-POSS materials appear to show an increased group delay at the 1-hour storage mark, and then a decrease near the original value. There does not appear to be any correlation between the attenuation and group delay data in the POSS systems. In contrast, in the POSS materials, as shown in Figure 14, both the PMMA and PS showed decreasing group delays with increased storage time, while that of the Bakelite-based low-k material was quite stable. The observations in the organic films were consistent with the changing dielectric properties. The changing dielectric properties originated from the gradual thermal decomposition of the materials.

### Implication of the thermal degradation on Low-k materials dielectric reliability studies

It is critical to understand the physics of failure and the electrical conduction mechanisms under bias stress and elevated temperatures,^57^ as the failure of the insulation of low-k and ultra-low-k dielectric materials in interconnects is still not fully understood. The data presented above show that unbiased low-k dielectric materials can thermally degrade at temperatures around 200°C, depending on the underlying chemistry. Lam et al.^58^ have also shown that at room temperature, under bias, intrinsic degradation of the dielectric material precedes metal ion out-diffusion into the dielectric. They suggest that energetic electrons injected from the cathode break chemical bonds, especially within the -Si-O-Si- network. This allows metal ions to diffuse into the dielectric. The changes in the chemical bonding and the subsequent ingress of metal ions result in stress and strain in the dielectric. Our data, when viewed in the context of Lam et al., suggest that at there are at least two contributions to the material damage that precede the electrical low-k material breakdown: electron-induced and thermal-induced bond cleavage. While the electron-induced damage appears to focus on the siloxane network, the thermal induced damage is centered around random scissoring of polymer backbone and silicoorganic (Si-C) bonds.

### Implication of thermal degradation of dielectric materials on high speed device performance

In high speed data transfer applications, the changing low-k properties, and group delay, could introduce resistive losses into the signal propagation in the interconnects. Experience has shown that lossy, unterminated transmission lines can be used successfully to propagate high-speed signals if the amount of loss is less than the design loss budget.^32^ However, accurate predictions of the loss budget require that all the loss mechanisms be accounted for in the models. As we have shown in this paper, the changes in the signal propagation characteristics of low-k materials are both temperature and frequency dependent. Hence the resistive losses stemming from the changing interconnect insulating material must be factored into the material selection process.

## Conclusions

We have leveraged changes in the microwave propagation characteristics to identify thermally-induced degradation in prototypical low-k thin films deposited on silicon substrates. Contrary to expectations, the thermally-induced irreversible chemical changes were observed in carbon doped low-k films stored at 200°C for at least one hour. These chemical changes result in increased microwave insertion losses in the interconnects, and translate into increased interconnect resistivity and changes in the group delay, which could have device performance consequences in high speed operations. Whereas it would have been useful to leverage the insights developed from this work to map out the prerequisites for thermally stable low-k materials, we are unable to do so because of our limited material processing knowledge.

The observations from this work extend the application scope of our non-destructive broadband RF-based metrology platform for understanding and quantification of the reliability issues associated with the back-end-of-the-line (BEOL) metallization and with emerging nanoelectronic devices. The RF-based platform is a promising technique that could allow us to gain insights into the thermal stability and reliability of emerging materials such as low- and ultra-low dielectric constant materials.

## Figures and Tables

**Table I:** Compendium of the known properties of the low-k dielectric samples studied in this work.

Sample ID	Process Type	CTE (ppm/C)	Film Density (g/cm^3^)	Porosity (%)	Dielectric Constant	Fraction % pores
A	SOD	23	0.74	42.2	2.3	74
B	SOD	22	0.88	43.2	2.3	3.9
C	CVD	-	1.25	18.2	2.8	-
D	CVD	67	1.24	11.2	2.5	71.5
E	SOD	-	1.35	14.6	-	-
F	CVD	58 ± 14	1.30	33	2.2	-

## Footnotes

a Certain commercial equipment, instruments, or materials are identified in this report in order to specify the experimental procedure adequately. Such identification is not intended to imply recommendation or endorsement by the National Institute of Standards and Technology, nor is it intended to imply that the materials or equipment identified are necessarily the best available for the purpose.
